# Abnormal (Hydroxy)proline Deuterium Content Redefines Hydrogen Chemical Mass

**DOI:** 10.1021/jacs.1c12512

**Published:** 2022-02-02

**Authors:** Hassan Gharibi, Alexey L. Chernobrovkin, Gunilla Eriksson, Amir Ata Saei, Zena Timmons, Andrew C. Kitchener, Daniela C. Kalthoff, Kerstin Lidén, Alexander A. Makarov, Roman A. Zubarev

**Affiliations:** † Division of Physiological Chemistry I, Department of Medical Biochemistry and Biophysics, Karolinska Institutet, SE-171 77 Stockholm, Sweden; ‡ Pelago Bioscience, SE-171 48 Solna, Sweden; § Department of Archaeology and Classical Studies, Stockholm University, SE-114 19 Stockholm, Sweden; ∥ Department of Cell Biology, Harvard Medical School, Boston, Massachusetts 02115, United States; ⊥ Department of Natural Sciences, National Museums Scotland, Chambers Street, Edinburgh EH1 1JF, U.K.; # Department of Zoology, Swedish Museum of Natural History, SE-104 05 Stockholm, Sweden; ∇ Thermo Fisher Scientific GmbH, 28199 Bremen, Germany; ○ Department of Pharmacological & Technological Chemistry, I. M. Sechenov First Moscow State Medical University, Moscow 119991, Russia; ◆ The National Medical Research Center for Endocrinology, Moscow 115478, Russia

## Abstract

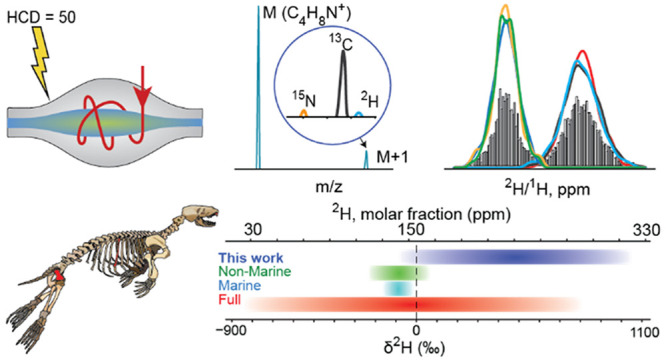

Analyzing the δ^2^H values in individual amino acids of proteins extracted from vertebrates, we unexpectedly found in some samples, notably bone collagen from seals, more than twice as much deuterium in proline and hydroxyproline residues than in seawater. This corresponds to at least 4 times higher δ^2^H than in any previously reported biogenic sample. We ruled out diet as a plausible mechanism for such anomalous enrichment. This finding puts into question the old adage that “you are what you eat”.

Deuterium (^2^H) is a stable isotope of hydrogen present in nature at the level of 120–150 ppm. Measurements of the deviations δ^2^H in deuterium content from the standard (ocean water, δ^2^H = 0‰) are widely used in science for terrestrial^1^ and extraterrestrial^2,3^ samples. An important biogenic source for δ^2^H analysis is collagen, the most abundant protein in bones and skin.^4^ Collagen molecules form strong fibrils that can survive thousands if not millions of years after an animal’s death^5^ because of the protective bone matrix.^6−8^ Deuterium content together with ^13^C and ^15^N abundances are used to derive information on the habitat and diet of the animals, some long extinct, as well as the climate in their lifetimes.^9−11^

Most of the collagen δ^2^H measurements that have been reported to date in the scientific literature fall into a limited δ^2^H range, usually ±100‰ (10%).^12^ The highest published δ^2^H values, ∼200‰, are found in bone collagen of adult marine piscivores.^12,13^ In 2008, a record 299‰ enrichment in collagen from a seal bone was reported in the Quoygrew medieval burial site on the Orkney Islands, Scotland.^14^

Here we found δ^2^H values in seal bone collagen that are several times higher than that, greatly surpassing the upper bound of hydrogen chemical mass recorded to date in any biogenic sample. Unlike the previous studies concerning bulk collagen, the reported extreme δ^2^H values are found only in two types of amino acid residues present in collagen, namely, proline (Pro) and its post-translationally modified derivative hydroxyproline (Hyp). Given that Pro and Hyp compose >22% of all residues in mammalian collagen and that they are roughly twice as heavy as glycine, the most abundant collagen amino acid, our result is in broad agreement with the 2008 measurements performed on bulk protein. Nevertheless, our finding represents a dramatic extension of the range of deuterium content in natural substances, redefining the biogenic chemical mass for this important element (Figure 1).

**Figure 1:**
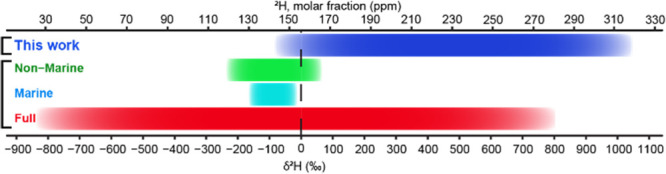
Deuterium range in nature according to IUPAC and its extension in this work.

Isotopic ratio measurements with amino acid resolution in collagen were performed using the recent method of Fourier transform isotopic ratio mass spectrometry (FT IsoR MS) (Figure 2A). In short, collagen is extracted from animal bones as in conventional isotopic ratio mass spectrometry (IR MS) and then digested by trypsin, as is common in proteomics. The obtained peptide mixture is analyzed in a proteomics-type data-dependent experiment with liquid chromatography coupled with tandem mass spectrometry (LC-MS/MS), with the only difference being that after the conventional MS/MS event generating sequencing information (which in FT IsoR MS is optional), an additional MS/MS event is introduced. This event uses a broad *m*/*z* window for precursor ion isolation followed by hard gas-phase collision-induced dissociation, fragmenting collagen tryptic peptide ions down to immonium ions (H_2_N^+^=CHR, where R represents the side chain of an amino acid residue). All aliphatic amino acids as well as aromatic ones give abundant immonium ions. For Pro and Hyp, the immonium ions are cyclic: C_4_H_8_N^+^ for Pro and C_4_H_8_NO^+^ for Hyp. The mass resolution of 60 000 at *m*/*z* 200 for the Orbitrap mass analyzer enables measurements of the “fine structure” M + 1 peak components for the isotopes ^2^H, ^13^C, and ^15^N (Figure 2B). The specific isotopic ratio (e.g., ^2^H/^1^H) is obtained from the FT IsoR MS/MS spectrum as the abundance ratio of a specific component (the ^2^H component in this Communication) of the fine structure of an M + 1 isotopic peak and the abundance of the monoisotopic peak M in a given tandem mass spectrum, and dividing this ratio by the number of atoms of the corresponding element in the immonium ion (in the case of hydrogen, seven for Pro ions and eight for Hyp ions). In an LC-MS/MS analysis of collagen peptides, several hundred individual isotopic measurements are obtained, forming a bell-shaped distribution (Figure 2C). The most probable value of this distribution provides the final δ^2^H result. The δ^2^H variation between replicate analyses is ≤3‰ in the case of abundant immonium ions, such as for Pro, Hyp, and Ile/Leu. To validate the FT IsoR MS method, we performed analysis of a proxy standard composed of free amino acids individually analyzed at the bulk level by two certified IR MS laboratories (Figure S1).

**Figure 2:**
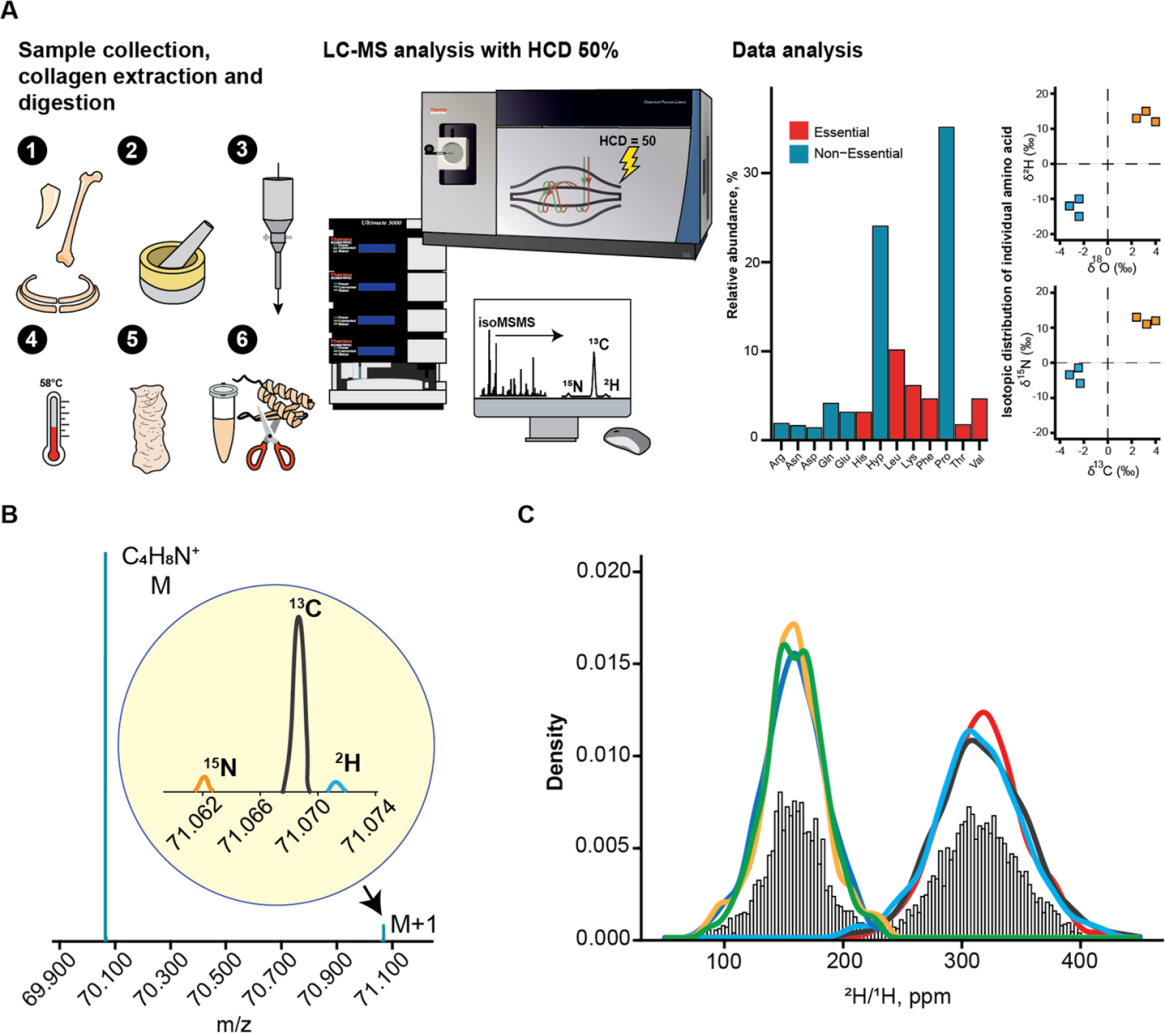
(A) Workflow of FT isoR MS: (1) bone sampling; (2) bone homogenization; (3) bone decalcification with HCl, (4): bone powder hydrolysis in diluted HCl at elevated temperature; (5) lyophilized collagen; (6) in-solution trypsin digestion followed by LC-MS/MS and data processing. (B) Monoisotopic M peak and “fine structure” M + 1 peak components for the ^2^H, ^13^C, and ^15^N isotopes of a Pro immonium ion. (C) Distributions of the ^2^H/^1^H ratios for Pro immonium ion obtained during a 1 h LC-MS/MS analysis of two distinct collagen samples (swan and gray seal) in three replicates each; the reported value is the mean of the average values from the replicate distributions.

The FT IsoR MS results for gray seal collagen in comparison with swan collagen (the latter was chosen as an interim standard) are shown in Figure 3A. In Pro immonium ion 
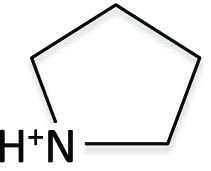
, the nitrogen-bound hydrogen is labile, and thus, the observed enrichment is diluted as 7/8 by the δ^2^H in the LC solvent, which was taken to have the ^2^H/^1^H ratio of 147 ppm. In Hyp immonium ions 
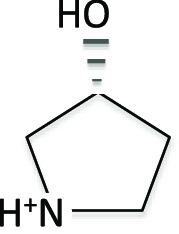
, two hydrogen atoms are labile, and thus, the dilution was 6/8. After correction was made for the dilution effect, the maximum δ^2^H values were 1209‰ for Pro and 1468‰ for Hyp. At the same time, for the Leu/Ile immonium ions a slight depletion (−6‰) was observed.

**Figure 3:**
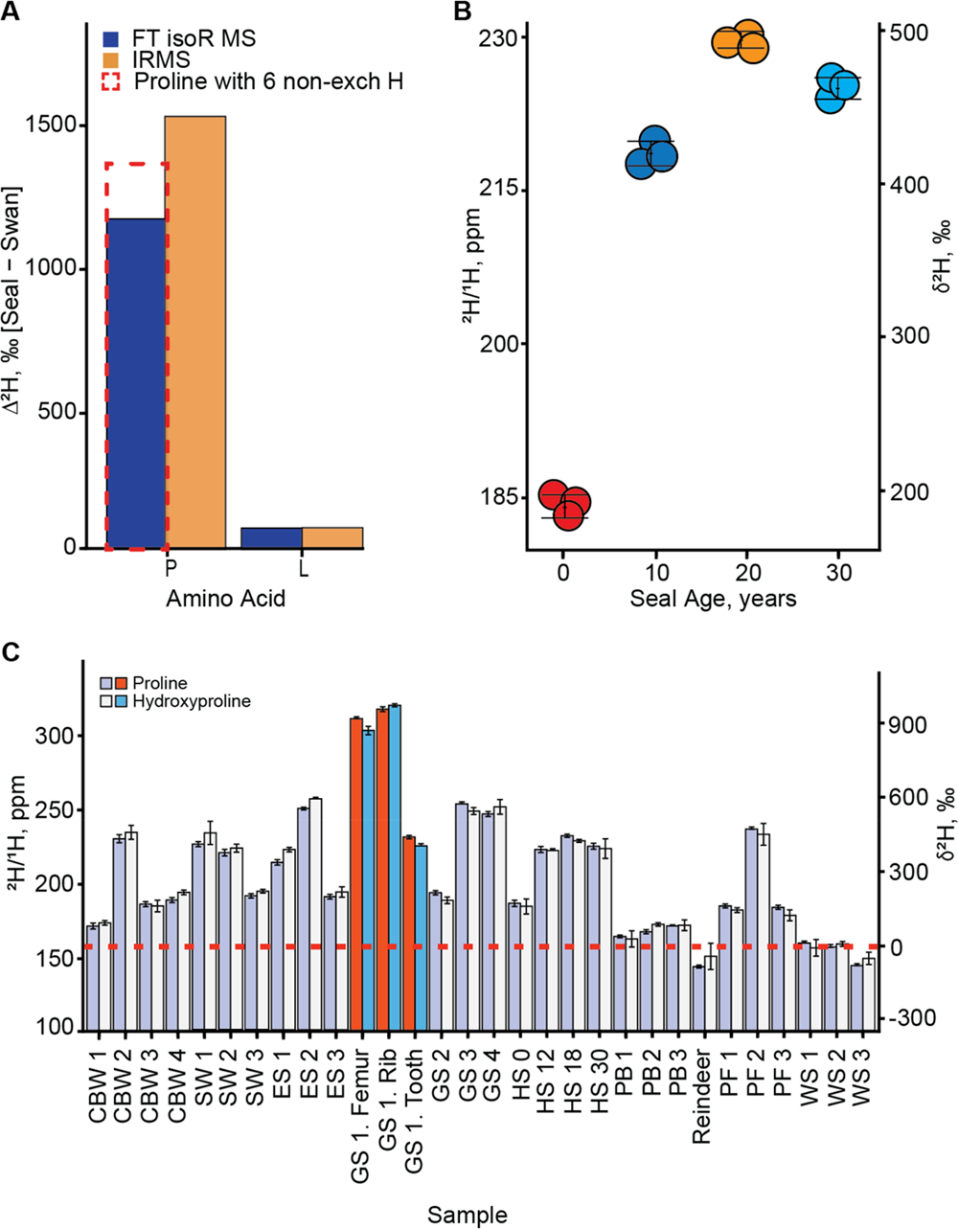
(A) Bar plot showing the δ^2^H difference between seal and swan samples in IRMS and FT isoR MS (The dashed plot is for a different number of labile hydrogens) for proline (P) and leucine (L). (B) Deuterium accumulation with age in four harbor seals. Each sample was analyzed in three replicates. (C) Deuterium contents in Pro and Hyp in analyzed samples: CBW = Cuvier’s beaked whale, ES = southern elephant seal, PF = peregrine falcon, GS = gray seal, HS = harbor seal, PB = polar bear, SW = sperm whale, WS = whooper swan.

These extraordinary results were verified by amino acid-resolved isotope ratio MS, in which collagen is first hydrolyzed to free amino acids that are then derivatized to become volatile, separated by gas chromatography, and combusted, with the resultant ^2^H analyzed by a magnetic sector mass spectrometer. The obtained δ^2^H value for Pro, 1700‰, corresponds to even higher enrichment than the FT IsoR MS value (Hyp was not analyzed). Of the 12 amino acids analyzed (Ala, Gly, Ser, Pro, Asp, Glu, Thr, Val, Leu, Ile, Phe, and Lys), all but Gly showed enrichment in seal versus swan, though none as much as Pro (Table S1). The second-most enriched amino acid, Thr, had a δ^2^H value >3 times lower than that of Pro.

We also used FT IsoR MS to analyze bone collagen from the bones of various animals collected in different geographical areas (Figure S2) dating from the medieval age until modern times and samples with different biological ages to have a more comprehensive analysis (Figure 3B,C). Many samples showed elevated deuterium levels in Pro and Hyp. For example, the fastest flying bird, the peregrine falcon (speeds up to 320 km/h^15,16^) showed δ^2^H values up to 500‰ (Figure 3C). Among different seal species, gray seals (*Halichoerus grypus*) from Scotland had the highest values (Figure 3C). There seems to be a tendency for δ^2^H values to increase with biological age in seals, although the data do not exclude saturation at puberty (Figure 3B). At the same time, in all of the analyzed species, Ile/Leu residues had unremarkable δ^2^H values near zero (Table S2).

Finding an explanation for this phenomenon turned out to be problematic. Pro is a nonessential amino acid in mammals, and it can be synthesized from both glutamate and ornithine.^17^ In IRMS analysis, however, Glu and Lys (the closest analogue of ornithine) were the fifth and ninth most enriched residues out of 12 (Table S1), which makes de novo biosynthesis of Pro an unlikely cause of deuterium enrichment.

Diet is also a doubtful explanation: in laboratory experiments on mice, δ^2^H in adult bone collagen was always below that of food.^18^ Also, δ^2^H values for Pro and Hyp in polar bears that feed mostly on seals were only slightly above those of the reindeer and much below those of the seals (Figure 3C).

Looking for other explanations, we considered radical reactions that can be initiated in proteins by mechanical stress.^19^ In structurally stressed peptides, the acidities of the α-proton are higher in prolyls than other amino acid residues abundant in collagen,^20^ and thus, the prolyl α-carbon is a likely site of hydrogen abstraction. As the C–D bond is stronger than the C–H bond,^21^ deuterium atoms at α-carbons should be less likely to be abstracted and more readily attached to α-carbon radicals than hydrogen atoms. However, when bone collagen was ground in a ball mill containing water, no significant alteration in proline deuterium was detected at different deuterium contents in water.

Another hypothesis to check was whether deuterium enrichment could be caused by stress during the growing phase of the collagen-producing cells. To test this possibility, we grew human fibroblasts under different stress conditions (water shortage, food starvation, and temperature fluctuations) for 6 weeks. None of the tested stress conditions caused deuterium enrichment in Pro exceeding the ^2^H content of the medium.

We further hypothesized that since proline side chains easily make complexes with metal(II) ions and such complexes undergo hydrogen–deuterium exchange at elevated temperature,^22,23^ the presence of these ions that stabilize collagen filaments in bones^24,25^ can lead to deuterium accumulation in proline. To test this hypothesis, we incubated collagen peptides with Zn^2+^, Cu^2+^, Mn^2+^, and Mg^2+^ salts at 90 °C for 72 h in water containing normal as well as elevated deuterium content. No enrichment in proline relative to δ^2^H in water was observed.

Remaining a mystery, the biochemical pathway for deuterium enrichment in Pro and Hyp residues of proteins calls for further investigation of this intriguing phenomenon.

## Supporting Information Available

Details of the mass spectrometric analyses used to generate the data and materials and methods (PDF)
