# Supplementary material for: Abnormal (Hydroxy)proline Deuterium Content Redefines Hydrogen Chemical Mass
Source: J Am Chem Soc. 2022 Feb 2;144(6):2484–7. doi: 10.1021/jacs.1c12512 (PMC8855419; doi:10.1021/jacs.1c12512)
Supplement: Supplementary file 1 — ja1c12512_si_001.pdf [file ja1c12512_si_001.pdf]

Supporting Information for

Manuscript title: Abnormal (hydroxy)prolines deuterium content redefines hydrogen chemical mass

Hassan Gharibi‡[1], Alexey L. Chernobrovkin‡[1,2], Gunilla Eriksson[3], Amir Ata Saei[1,4], Zena Timmons[5], Andrew C. Kitchener[5], Daniela C. Kalthoff[6], Kerstin Lidén[3], Alexander A. Makarov[7], and Roman A. Zubarev*[1,8,9]

[1]Division of Physiological Chemistry I, Department of Medical Biochemistry and Biophysics, Karolinska Institutet, Stockholm, Sweden, SE-171 77

[2]Pelago Bioscience, Solna, Sweden, SE-171 48

[3]Department of Archaeology and Classical Studies, Stockholm University, Stockholm, Sweden, SE-114 19

[4]Department of Cell Biology, Harvard Medical School, Boston, U.S., MA 02115

[5]Department of Natural Sciences, National Museums Scotland, Chambers Street, Edinburgh EH1 1JF, UK

[6]Department of Zoology, Swedish Museum of Natural History, Stockholm, Sweden, SE-104 05

[7]Thermo Fisher Scientific GmbH, Bremen, Germany, DE 28199

[8]Department of Pharmacological & Technological Chemistry, I.M. Sechenov First Moscow State Medical University, Moscow, Russia, RU 119991

[9]The National Medical Research Center for Endocrinology, Moscow, Russia, RU 115478

Table of content

Materials and Methods 3

Tables 5

Figures 7

References 9

Materials and Methods

Materials

Sequencing Grade Trypsin (Promega, U.S.), HyperSep™ Filter Plates (Thermo Scientific, U.S.), Ammonium bicarbonate ReagentPlus® (Sigma-Aldrich, U.S.), PepMap

Methods

Sample preparation and collagen extraction

Pieces of bones were collected from different sources (Stockholm University, Museum of Natural History Stockholm (NRM), and National Museums Scotland), and collagen extraction was performed according to Brown's[1] protocol. Collagens then were weighted and approximately 200 µg protein was dissolved in 50 mM Ammonium Bicarbonate buffer at 70 °C for 3 hours. Samples were centrifuged at 14000 g for 10 minutes and then supernatant of dissolved collagen was transferred to a new 1.5 ml Eppendorf tube. Sequencing Grade trypsin was used as the enzyme to break the protein to peptides during the digestion process. Trypsin solution was added to each sample with the ratio of 1:50 (enzyme to protein) and incubated overnight at 37 °C in a Heating Block mixing with 300 rpm rate. The enzymatic reaction was quenched by adding up to 5% Formic Acid (FA). Digested proteins (peptides) were desalted using HyperSep™ Filter Plates. Peptides were dried in a speed vac instrument overnight. The dried peptides were dissolved in buffer A (98% Water, 1.9% Acetonitrile, and 0.1% FA) to the 0.5 µg/µl concentration.

FT IsoR MS

Samples were loaded with buffer A (0.1% FA in water) onto a 50 cm EASY-Spray column (75 µm internal diameter, packed with PepMap C18, 2 µm beads, 100 Å pore size; Cat#ES803A) connected to the UltiMate™ 3000 RSLCnano (Thermo; Cat#ULTIM3000RSLCNANO) and eluted with a buffer B (98% ACN, 0.1% FA, 2% H2O) gradient from 4 to 15% of at a flow rate of 300 nL/min for 50 minutes and then increased to 35% for 10 minutes and washed at 95% for 7 minutes. Mass spectra were acquired with an Orbitrap Fusion Lumos Tribrid Mass Spectrometer (Thermo; Cat# IQLAAEGAAPFADBMBHQ) in the data-independent mode with targeted MS^2 selecting peptide with m/z equal to 800 (doubly charged) and 1000 m/z isolation window and scan at 60,000 (@200 m/z) resolution, in the m/z range from 50 to 200. Peptide fragmentation was performed via higher-energy collision dissociation (HCD) with energy set at 50 NCE.

Conventional Isotopic Analysis with Isotopic Ratio Mass Spectrometry (IRMS)

All samples were analyzed at the Edge Institute, University of California, Riverside. The $\delta^{13}C$ and $\delta^{2}H$ values of individual amino acids were determined by gas chromatography/combustion/isotope ratio mass spectrometry using a Trace 1310 gas chromatograph (GC) coupled with a GC-Isolink ll routed through a Conflo IV interface into a Delta V Plus isotope ratio mass spectrometer (Thermo Fisher Scientific, USA). Amino acids were either routed through a combustion reactor at 1000 °C ($\delta^{13}C$) or an HTC reactor at 1420

°C (δ^2H). Using a Triplus RSH autosampler, samples were injected splitless at 250 °C onto a BPX-5 capillary column (60 m x 0.32 mm inner diameter, 1.0 um film thickness; SGE Analytical Science, USA). The GC oven temperature program was: initial temperature 50 ℃ for 2 minutes, followed by a ramp of 15 °C/min to 125 °C, a ramp of 3 °C/min to 160 °C, a ramp of 4 °C/min to 190 °C, a ramp of 6 °C/min to 275 °C, and a ramp of 15 °C/min to 320 °C. Carrier gas (He) flow was 2 ml/min.

Data Analysis

The in-house ISOMS package was used to do the isotopic analysis on immonium ions fragments. The MSConvert (version 3.0.20168) from ProteoWizard Tools (http://proteowizard.sourceforge.net/) was used to convert the mass spectrometer raw file to mzml format and then the in-house ISOMS (https://github.com/RZlab/isoms) package was used to convert the mzml files to a readable table in csv format.

Tables

Table S1. The obtained δ^{2}H for 12 amino acids in Grey Seal 1 Rib and Whooper Swan 1 samples from IRMS instrument.

Amino Acid	δ^{2}H, ‰ (with IRMS)		
Sample	GS 1 Rib	WS 1	Difference (Seal vs Swan)
Ala	-10.2	-92.1	81.9
Gly	-157.9	-130.6	-27.3
Ser	-11.7	-193.5	181.8
Pro	1700.0	194.8	1505.2
Asp	48.0	-117.3	165.3
Glu	5.9	-96.8	102.7
Thr	305.2	-159.1	464.3
Val	-121.9	-196.3	74.4
Leu	-97.9	-159.7	61.8
Ileu	-111.9	-200.4	88.5
Phe	-89.7	-157.4	67.7
Lys	-44.1	-115.2	71.1

Table S2.

The recorded δ^{2}H by FT IsoR MS method across all samples listed in Fig.3. SD is the standard deviation and n is number of replicates.

Amino Acid	Sample	δ^{2}H, ‰	SD	n
Leu	CB 1	5.7	37.5	3
Leu	CB 2	9.8	23.6	3
Leu	CB 3	24.4	85.3	3
Leu	CB 4	32.2	24.3	3
Leu	SW 1	-51.9	52.1	3
Leu	SW 2	-42.2	21.8	3
Leu	SW 3	-67.2	10.7	3
Leu	ES 1	82.6	99.8	3
Leu	ES 2	5.3	32.4	3
Leu	ES 3	67.0	58.9	3
Leu	GS 1 Tooth	-78.4	21.2	3
Leu	GS 1 Femur	-112.9	49.2	3
Leu	GS 1 Rib	-62.2	62.5	3
Leu	GS 2	-72.3	26.4	3
Leu	GS 3	-84.4	35.3	3
Leu	GS 4	-120.8	32.8	3
Leu	HS 0	-59.7	46.2	3
Leu	HS 12	-70.0	73.6	3
Leu	HS 18	-97.0	41.7	3
Leu	HS 30	-79.4	63.3	3
Leu	PB 1	-6.9	36.2	3
Leu	PB 2	101.6	50.6	3
Leu	PB 3	-121.8	23.6	3
Leu	PF 1	-116.2	33.6	3
Leu	PF 2	-78.3	4.3	3
Leu	PF 3	-115.6	53.4	3
Leu	WS 1	-107.9	41.4	3
Leu	WS 2	-52.7	45.8	3
Leu	WS 3	-108.0	30.9	3
Leu	Reindeer	-15.7	65.3	3

Figures

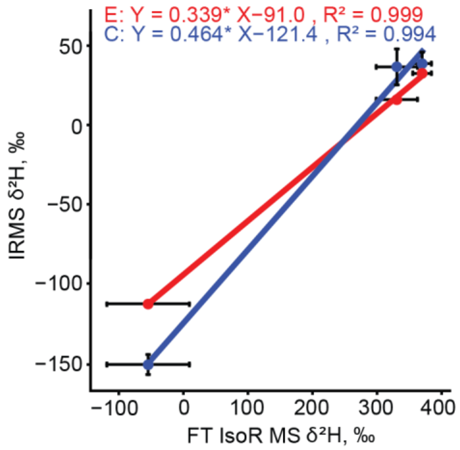

Figure S1. FT IsoR MS validation with IRMS. To validate the FT IsoR MS method we preformed analysis of three free amino acids (Proline, Hydroxyproline, and Valine) individually by two certified IRMS laboratories (E: Elemtex Laboratory, UK. C: Stable Isotope Lab, Cornell University, US.)

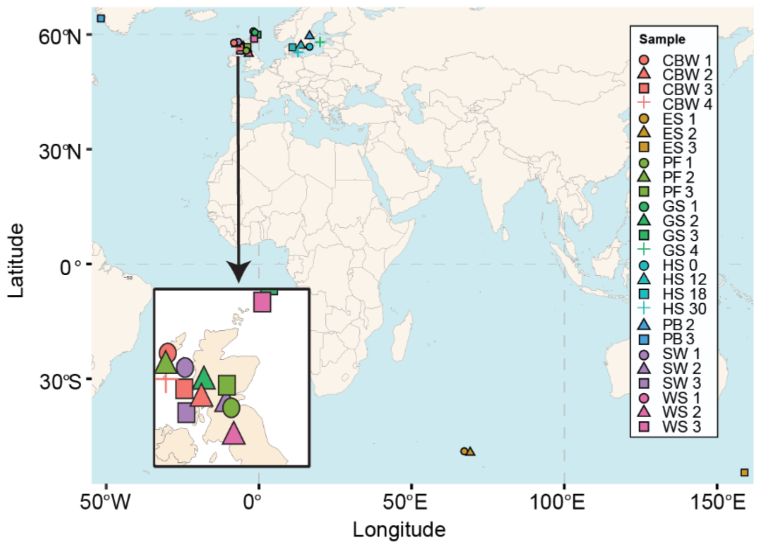

Figure S2.

Map for the collected samples, CBW = Cuvier's Beaked Whale, ES = Southern Elephant Seal, PF = Peregrine Falcon, GS = Grey Seal, HS = Habour Seal, PB = Polar Bear, SW = Sperm Whale, WS = Whooper Swan.

References

(1) Brown, T. A.; Nelson, D. E.; Vogel, J. S.; Southon, J. R. Improved Collagen Extraction by Modified Longin Method. *Radiocarbon* **1988**, *30* (2), 171–177. https://doi.org/10.1017/s0033822200044118.
